# First Results of Using a UVTron Flame Sensor to Detect Alpha-Induced Air Fluorescence in the UVC Wavelength Range

**DOI:** 10.3390/s17122756

**Published:** 2017-11-29

**Authors:** Anita J. Crompton, Kelum A. A. Gamage, Steven Bell, Andrew P. Wilson, Alex Jenkins, Divyesh Trivedi

**Affiliations:** 1 Engineering Department, Lancaster University, Lancaster LA1 4YW, UK; 2 School of Engineering, University of Glasgow, Glasgow G12 8QQ, UK; kelum.gamage@glasgow.ac.uk; 3 Nuclear Metrology Group, National Physical Laboratory, London TW11 0LW, UK; steven.bell@npl.co.uk; 4 Independent researcher, Warrington WA5 9YX, UK; andy.wilson1962@talktalk.net; 5 Characterisation, Inspection & Decontamination Group, Sellafield Ltd., Cumbria CA20 1PG, UK; alex.jenkins@sellafieldsites.com; 6 The National Nuclear Laboratory, Warrington WA3 6AE, UK; divyesh.trivedi@nnl.co.uk

**Keywords:** UVTron flame detectors, alpha detection, alpha-induced air fluorescence, alpha imaging, nuclear decontamination and decommissioning

## Abstract

In this work, a robust stand-off alpha detection method using the secondary effects of alpha radiation has been sought. Alpha particles ionise the surrounding atmosphere as they travel. Fluorescence photons produced as a consequence of this can be used to detect the source of the alpha emissions. This paper details experiments carried out to detect this fluorescence, with the focus on photons in the ultraviolet C (UVC) wavelength range (180–280 nm). A detector, UVTron R9533 (Hamamatsu, 325-6, Sunayama-cho, Naka-ku, Hamamatsu City, Shizuoka Pref., 430-8587, Japan), designed to detect the UVC emissions from flames for fire alarm purposes, was tested in various gas atmospheres with a ^210^Po alpha source to determine if this could provide an avenue for stand-off alpha detection. The results of the experiments show that this detector is capable of detecting alpha-induced air fluorescence in normal indoor lighting conditions, as the interference from daylight and artificial lighting is less influential on this detection system which operates below the UVA and UVB wavelength ranges (280–315 nm and 315–380 nm respectively). Assuming a standard 1r2 drop off in signal, the limit of detection in this configuration can be calculated to be approximately 240 mm, well beyond the range of alpha-particles in air, which indicates that this approach could have potential for stand-off alpha detection. The gas atmospheres tested produced an increase in the detector count, with xenon having the greatest effect with a measured 52% increase in the detector response in comparison to the detector response in an air atmosphere. This type of alpha detection system could be operated at a distance, where it would potentially provide a more cost effective, safer, and faster solution in comparison with traditional alpha detection methods to detect and characterise alpha contamination in nuclear decommissioning and security applications.

## 1. Introduction

The ability to detect alpha emissions from nuclear materials is important in nuclear operations, nuclear decommissioning, and nuclear security applications. Due to their positive charge, alpha particles travel only a short distance after emission from nuclear materials, typically around 50 mm through air depending on their energy. This is due to their interaction with atoms in the surrounding atmosphere. Therefore, detectors which require direct contact with alpha particles need to be in close proximity to any surface or object to determine if alpha contamination is present, at a distance of less than the mean free path of the alpha particles. This causes a number of issues, as documented by other researchers [1,2,3]. As objects to be monitored may be in a mixed radiation environment, personnel carrying out detection activities may require personal protective equipment (PPE) and have limited time in which they can safely operate. Large structures or complex geometries take significant time to monitor in such close proximity. A stand-off detector, where a significant distance between the detector and surface can be achieved, can reduce the time taken for monitoring whilst distancing the operator from the radioactive environment.

As an alpha particle travels it ionises the air, transferring energy to the atoms and molecules in its path, mostly due to its positive charge. These excited atoms may emit an optical photon to return to a stable state and these photons have a mean free path in the order of kilometres [4,5]. So in theory they could be detected from a significant distance, much further than direct alpha particle interaction detectors could achieve.

Although there have been experiments and several prototype detectors which have utilised this effect for stand-off alpha detection [1,6,7,8,9,10], there are significant difficulties with this approach. The main issue is the interference of light (natural or from lighting equipment), which is typically present at a much greater intensity compared with the signal from alpha-induced fluorescence. Operating in dark or special lighting conditions alleviates this problem somewhat, as can be seen by the success of Sand et al.’s detector system under special lighting conditions [7], where UV-free lighting sufficient to see was used to operate the equipment with a filtered photomultiplier tube PMT. Although where special lighting can be used this detector has positive results, it is not possible to always specify lighting conditions in the field. The variability of lighting levels over short time periods, especially from natural light sources, means that subtracting the background is a very difficult option and although a patent has been filed for such a detector [11], this requires the use of 6 separate cameras sensitive at 6 different wavelengths, and details on the testing of such a device are absent in the available literature. Although filtering can be used, it causes an unwanted attenuation of the desired signal along with the undesired background light, especially in the ultraviolet (UV) wavelength range, and special materials are required, for example fused silica. Although Sand et al. use filtering of a specific bandpass which coincides with a peak of 334 nm, the main peak from nitrogen fluorescence, they still require special lighting conditions to determine the signal from the background. Ivanov et al. had some success in using a solar-blind CCD (charge coupled device) camera [12] which could image UV fluorescence in daylight without the need for specific filtering. However, this is limited by the time taken to carry out imaging, process it, and then analyse the results, meaning that a solar-blind CCD camera would have limited applications in the field or for scanning purposes. Although there are ‘solar-blind’ detectors available, for example the Hamamatsu R7154P high sensitivity solar-blind photocathode (160–320 nm), upon testing this proved not to be completely solar blind, although it was much less sensitive above 320 nm. Hence, these are still affected by background light and not sensitive enough to determine the alpha-induced fluorescence from background in normal lighting conditions.

Therefore, this research has focused on a detector which is less affected by natural and artificial light than other stand-off detectors available to date: the UVTron by Hamamatsu. This detector is designed to detect UVC emissions from flames for use in fire detection systems and to have a negligible background count in normal indoor lighting. Experiments were carried out to determine if the UVTron was able to detect the UVC fluorescence photons generated by an alpha source, and the effect of different gas atmospheres on this detection.

## 2. Materials and Methods

**Figure 1:**
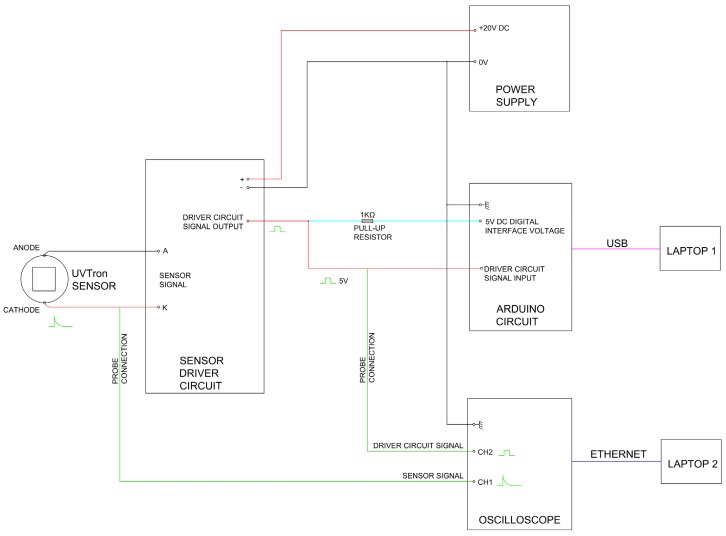
Schematic of equipment set-up.

Experiments were carried out at the National Physical Laboratory in Teddington, Middlesex. The set up for the experiments was as follows (see Figure 1).

**Figure 2:**
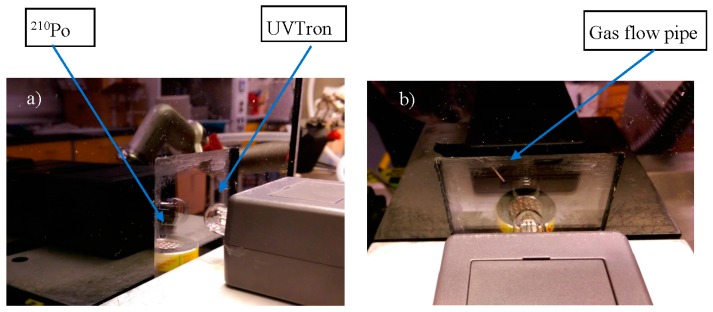
Photographs (**a**,**b**) showing the ^210^Po source inside the gas flow box (silver disk with mesh surface and yellow edge) and the UVTron (small glass bulb), attached to the grey box housing the detector electronics. In photograph (**b**) the tube through which the gas was flowed over the source can be seen to the left and above the source.

A ^210^Po source of 6.95 MBq (see Section 2.2, Source) was placed in close proximity to the UVTron detector (Hamamatsu, R9533, see Section 2.1, Detector), with an approximate separation of 20 mm between the source and detector, unless stated in the results. Experiments were run with the source inside a gas flow box with a fused silica window (see Section 2.3, Gas Flow Box), unless stated in the results (Figure 2).

The output from the UVTron was recorded directly using an oscilloscope (Infiniium 54845A, 8 GSa/s, 1.5 GHz, (Keysight Technologies, Santa Rosa, CA, USA) which recorded the shape of the direct pulse. The output of the UVTron driver (C10807, Hamamatsu, Hamamatsu City, Japan) was connected to an Arduino Uno which counted the number of output pulses per second. A second channel on the oscilloscope was also connected to the output of the driver circuit which recorded the time and shape of the driver output pulse. Hence the direct pulse from the UVTron and the processed pulse from the UVTron driver circuit could be compared.

Where the effect of gas atmosphere was tested, the gas flowed across the surface of the source from a pipe placed above the source (see Figure 2b). The gas flow rates are stated for each gas tested in Section 2.5.

The lab in which the experiments were carried out had no windows and conventional strip lighting. This lighting remained on for the duration of all experiments.

### 2.1. Detector

**Figure 3:**
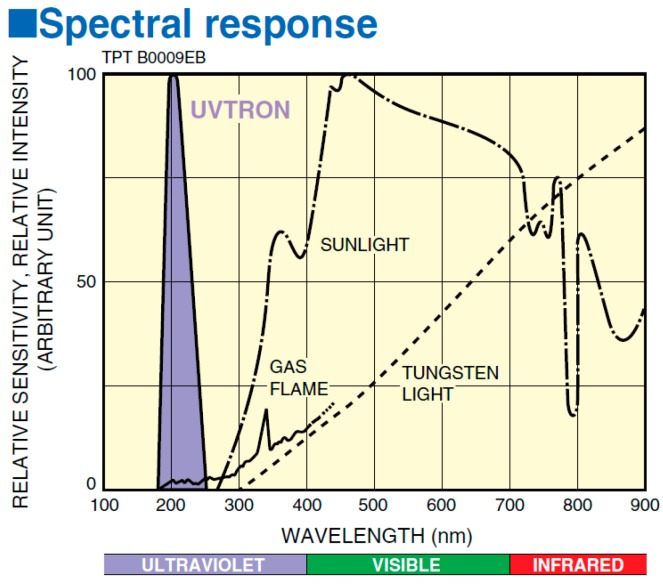
Spectral response of UVTron in comparison to sunlight, tungsten light, and gas flame [13].

UVTron detectors utilise the photoelectric effect and gas multiplication to generate an output pulse when a photon is incident on the photocathode. The UVTron used in this research (the R9533) has a Ni cathode which is insensitive to photons with a wavelength of greater than 260 nm. This makes it effectively solar blind (see Figure 3).

Photons within the 180–260 nm range when incident on the photocathode of the UVTron cause an electron to be emitted through the photoelectric effect. An electric field generated by a voltage differential between the cathode and the anode causes this free electron to be accelerated through the gas contained within the UVTron. As the negatively charged electron passes through the gas it interacts with the gas molecules and transfers energy, causing ionisation and creating ion pairs. The gas atmosphere within the UVTron bulb enhances this ionisation process. The created positive ions are attracted to the cathode, whilst the negative free electrons are attracted to the anode. As each free electron is generating further free electrons there is an avalanche reaction which causes an exponential increase in the number of free electrons incident on the anode. This generates a signal which is interpreted by the driver circuit and a pulse is outputted by the driver circuit when this avalanche phenomenon occurs.

The detector used for these experiments, R9533, is one example of one model in the UVTron range. It was selected due to its robust nature and the availability of an optimised off-the-shelf driver circuit.

### 2.2. Source

A sealed ^210^Po source was selected for these experiments as ^210^Po decays through alpha emission only, with a very low X/γ emission intensity (see [14] for a ^210^Po nuclide table). This was to eliminate the possibility of other radiations from the source generating a response from the UVTron, either through direct interaction or secondary effects. This source had an activity level of 6.95 MBq at time of use (29 August 2017). The source can be seen in the photographs of Figure 2.

### 2.3. Gas Flow Box and Gas Flow Set-Up

In order to provide a support for the source and to allow the testing of different gas flows, a box of dimensions 260 × 234 × 230 mm was constructed of 5-mm-thickness black Perspex. The box size was determined by the dimensions of an optional hemispherical reflector, which was not used in these experiments as the signal was sufficient for the detector to record an output.

**Figure 4:**
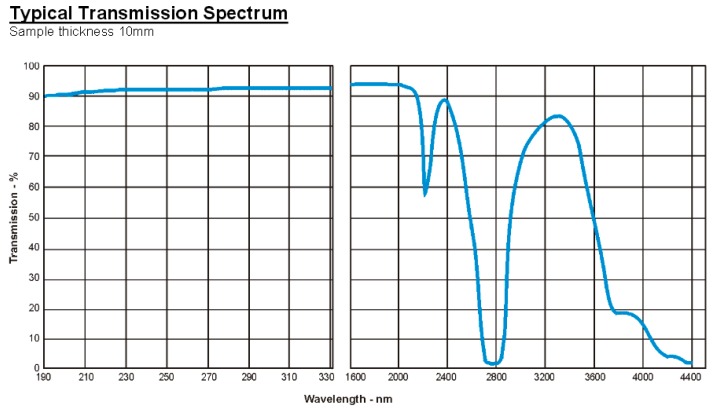
Transmission spectrum of fused silica window material [15].

The box has a 75 × 75 mm window of 2-mm-thick synthetic fused silica (Spectrosil^©^) (see Figure 2). Fused silica is preferable to ordinary glass as it allows UVC to pass with minimal attenuation (see Figure 4). This material is specifically designed for deep UV (UVC) optical applications.

The lid was placed on the box during gas experiments, but does not provide a gas tight seal. The gas was flowed over the source in an air atmosphere using a small flexible pipe of 1 mm bore diameter (see Figure 2b).

### 2.4. Measurement

Counts were made of the pulses per second emitted by the UVTron device driver (Hamamatsu C10807), which both powers the UVTron and processes the output signal. The UVTron driver outputs a 5-V square wave on each detected avalanche event from the UVTron, and the number of these per second was counted by the Arduino. The Arduino is unable to count the pulses directly from the UVTron as they are of 10-µs duration, too short for the Arduino to detect. Therefore, an oscilloscope was used to verify that each pulse directly from the UVTron was accompanied by a square wave pulse from the driver circuit. It was then manually verified by observation that each of these pulses correlated to a count from the Arduino. Hence, the counts per second of the Arduino were verified. The time delay between the two pulses was recorded for comparison to outputs using other UVC sources (e.g., UVC bulb, flame).

The direct pulses from the UVTron were recorded via the oscilloscope in order that pulse characteristics, i.e., shape, height, duration, could be determined and any difference between those resulting from an alpha source as compared to a UVC bulb or other UVC source could be identified. The 5-V square pulse from the UVTron driver circuit was also recorded using a second channel on the oscilloscope. The oscilloscope was set to a sample rate of 20 MHz, and 1800 acquisition points, with a trigger level of 6 V to ensure that the direct UVTron pulse which was approximately 12 V triggered each recorded event, rather than the UVTron driver which outputs a pulse of 5 V. However, both pulses were displayed and recorded as the trigger determines the time at which the output is measured for all channels in use (one for the direct UVTron output, the other for the driver output).

### 2.5. Gases

In order to determine the effect of different gases on the fluorescence count, five different gases were flowed over the source. The gases are as listed in the table below (Table 1):

In the field it may not be possible to flood the area under scrutiny with a specific gas, and in those environments which could provide a gas tight atmosphere, the amount of gas could be reduced by targeting the gas at the surfaces. Hurst et al. [16] in their experiments using protons to excite gases, found that it was preferable for the gas to flow through the gas cell they used rather than having a static gas atmosphere, as the former increased the amount of light intensity. The effects of various gases on alpha-induced fluorescence have been investigated [7,16,17,18,19,20]. However, to address the suitability for field operations and in light of Hurst et al.’s findings, a flow of gas over the source was tested. As the alpha particles travel in the order of 50 mm, the immediate surrounding area may prove to be more important in generating fluorescence photons than flooding the entire surroundings.

Following each experiment, the lid was removed and the gas vented via the fume hood extractor system. The supply pipe was flushed with the new gas. The lid was then replaced and the flow allowed to stabilise before the count was initiated.

### 2.6. Verification of Signal Source

In order to verify that the signal being received by the detector was indeed air fluorescence and not due to other emissions from the source, a number of checks were carried out. The set-up was as detailed above in Figure 1, with the source inside the gas flow box and the detector in close proximity to the fused silica window. An optical black-out cloth was placed between the source and detector. The observed counts per second (cps) immediately reduced to zero, returning to its former value when the cloth was removed. A piece of ordinary white paper was then placed between the source and detector, which also reduced the count to zero. Again, this returned to the former value once the paper was removed.

A sheet of fused silica glass of the same specification as the gas flow box window was placed between the source and the detector. This resulted in a small drop in cps of approximately 0.6%. This is broadly in line with the manufacturer’s specification, but not statistically significant due to counting uncertainties of ~2%.

As the signal was transmitted through the fused silica windows, but not through the paper or black-out cloth, a verification was made that the detector was detecting UVC photons from the alpha source and not X/γ/β radiation.

## 3. Results

### 3.1. Background Count

The UVTron detector and associated equipment was set up as per Figure 1. The lighting in the lab remained on for the duration of the experiment. Lighting in the lab consisted solely of strip (fluorescent) lighting as generally found in commercial buildings.

The background count was taken for a duration of approximately 75 min. Over this period a total of 10 pulses were recorded by the Arduino from the UVTron driver. This gives an average pulse rate of 2.224 × 10^–3^ ± 0.7034 × 10^–3^ counts per second. The pulses were randomly spread out over the time period and did not show any pattern.

The UVTron is subject to an extremely low background count and this had negligible effect on measurements taken using the alpha source, accounting for less than 1% of the detected signal for all gas environments investigated.

Several background counts have been made in different indoor locations and gave a range of results from 1.5 × 10^–3^ ± 0.3 × 10^–3^ to 3.6 × 10^–3^ ± 0.72 × 10^–3^ cps. All counts were carried out indoors. When in close proximity to a double-glazed window, the exterior lighting affected the count. As standard glazing attenuates UVC, it is likely that background counts outdoors will be higher.

For normal operation as a flame detector this value is negligible. For the detection of UVC from an alpha source, where the signal is far less intense than from a flame, this will be a limiting factor to the minimum sensitivity of the detector.

### 3.2. Air Atmosphere Results

Using the detector and associated equipment set up as in Figure 1, the ^210^Po source was placed inside the gas flow box in close proximity to the fused silica window. The UVTron detector was placed outside the box close to the fused silica window (see Figure 2). There was an approximate distance between the source and detector of 20 mm. With the lab lighting remaining on for the duration of the experiment, the output pulses of the UVTron driver circuit were counted by the Arduino.

**Figure 5:**
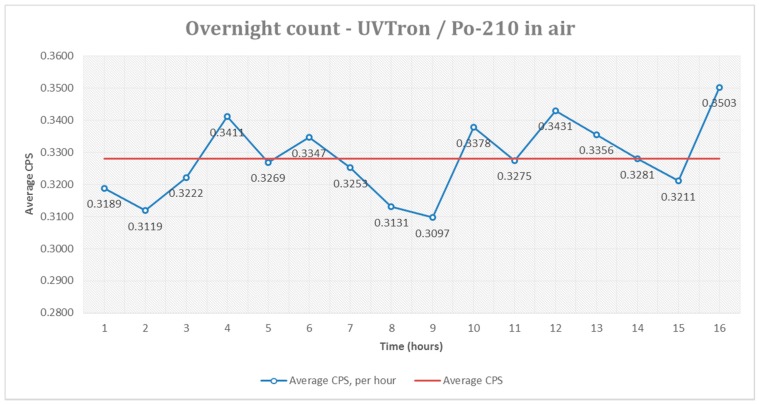
Average counts per second (cps) per hour—^210^Po source in air.

Over the 16 hours of the experiment 18,890 pulses were counted, giving an average of 0.3280 counts per second. This average varied over the duration of the experiment, with average hourly cps measured between 0.3097 cps and 0.3503 cps. Figure 5 shows the hourly average cps, compared with the overall average. This variation may have been due to changes in the environment over the 16-hour period, for example pressure in the lab (which varied between 1015 and 1010 mbar), humidity, temperature, etc., which may have affected UVC absorption or detector response.

The variability of the cps will also be a limiting factor in the minimum sensitivity of the UVTron.

### 3.3. Gas Flow Results

Five different gases were flowed over the source and the fluorescence measured was compared to an air atmosphere. These were selected following research into gases which were both likely to ionise at suitable energies and to emit photons of the required wavelength [16,17,18,19,20,21,22,23]. Although several of the studies used focus on ionisation of gases [21,22,23], this leads to secondary electrons which are believed to generate the fluorescence photons [16]. Saito et al. [20] identified krypton, xenon, and argon as fluorescing in the UVC wavelength range. Using the evidence from Saito and the other literature, nitrogen, argon, xenon, krypton, and neon were selected for testing. Argon was not available at the time of the experiment, however, there was an available supply of P-10 which was included in the testing due to its ionising properties.

**Figure 6:**
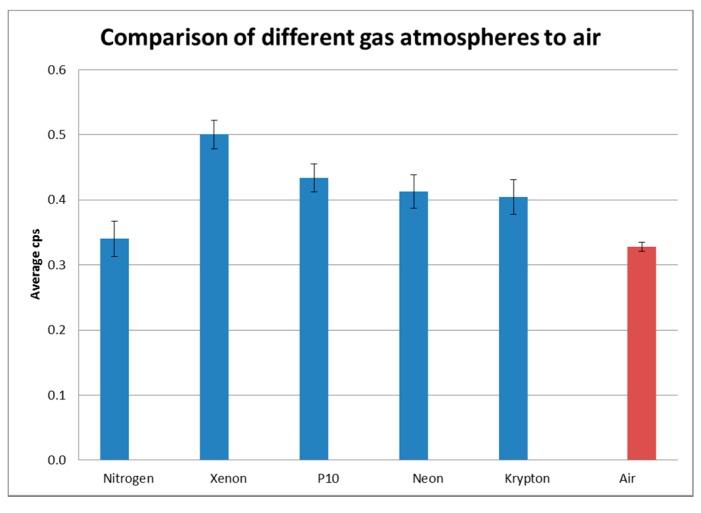
Comparison of average cps in different gas atmospheres.

The gas was flowed over the source from a distance of approximately 30 mm. The gases and flow rates are detailed in Section 2.5, Table 1. Each experiment was carried out for a duration of no less than one hour to provide a counting uncertainty below 3%. The results are shown in Figure 6 and in Table 2.

As can be seen from Table 2, there was an increase in the average cps for all gases compared to the air atmosphere. Xenon provided the greatest effect, with a 52% increase in the average cps compared to air. This finding is most significant as an increase in the signal will make detection easier, and the ability to replicate this in the field could greatly enhance detection possibilities.

P10 (10% methane, 90% argon) is used in gas-filled ionising radiation detectors due to its ionising properties, and was tested to determine if these properties would be beneficial in this application. It increased the cps by 32% compared to the air atmosphere. Neon and krypton also showed a significant increase in the cps recorded, 26% and 23% respectively.

Nitrogen showed only a small increase, which could be accounted for within the uncertainty and therefore may not be an actual observed increase. As this is the primary constituent of air and it is known to enhance fluorescence in the 300–400 nm wavelength range [1,2,5,6,8,9] this result was unexpected. Sand et al. [7] found an increase in fluorescence in the deep UV (around 260 nm) using a nitrogen purge. As Brett et al. [17] found there was a lack of fluorescence in the 230–290 nm wavelength range in air as compared to a pure nitrogen atmosphere, which they attributed to oxygen quenching. In this instance, using a flow of gas as opposed to a fully purged gas atmosphere would appear to have a great effect on fluorescence. For field operations the possible effect of oxygen quenching on the gases which showed a marked increase in fluorescence in these tests may merit further investigation, and that a repeat of the experiments to determine replication possibilities in the field be carried out.

### 3.4. Pulse Shape

The oscilloscope was used to record the pulse shape from each of the experiments in order that any differences could be observed and the pulse could be compared to pulse shapes from other UVC emitting sources, such as a UVC-emitting bulb, flame, or from a different gas atmosphere. Although the design of the detector rather than the source is most likely to determine the output pulse, differences would provide a valuable avenue for determining the source of the UVC photons and therefore increase the potential of a detector in the field. A minimum of 50 pulses were recorded for each experiment for comparison.

**Figure 7:**
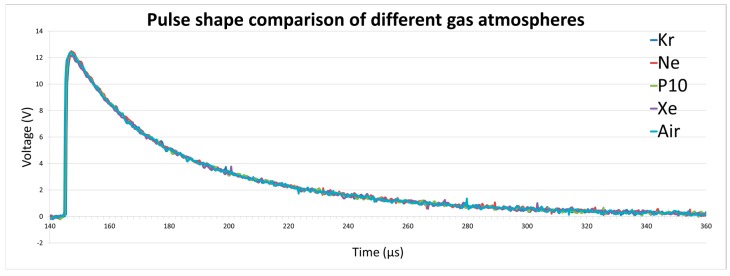
Pulse shape comparison of different gas atmospheres.

For each of the experiments carried out with the ^210^Po source, the pulse shape showed no distinguishable difference between different gas atmospheres. Figure 7 shows a comparison of the shapes of a single pulse recorded during each of the gas experiments. From this it can be seen that it is not possible to differentiate one gas from the other in terms of the pulse shape. Therefore, it can be concluded that different gas atmospheres do not affect the characteristics of the pulse outputted by the UVTron.

## 4. Conclusions

These experiments show that it is possible to detect alpha-induced air fluorescence using the UVTron flame detector in normal lighting conditions.

**Figure 8:**
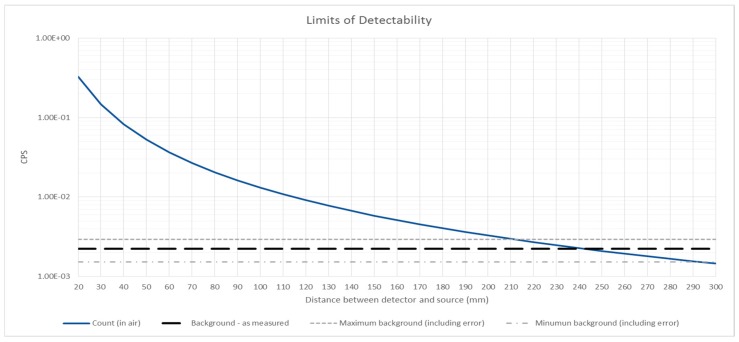
Limits of detectability showing the drop off in signal due to increased distance between sensor and source. The recorded background count ± the calculated error is shown.

Assuming a standard 1r2 relationship for the drop off in signal with distance (r) between the source and detector, the limit of detectability, based on the signal being above the level of background, is approximately 240 mm, as shown in Figure 8. This data is for the 6.95 MBq source with the R9533 UVTron sensor. This may be the potential limit of this particular configuration, however it is much greater than the distance of alpha particle travel and indicates that this method has potential for stand-off detection through further work.

It is also possible to estimate the minimum detectable activity for this sensor. Assuming a source of equal properties to the ^210^Po source of 6.95 MBq, excepting a reduced activity level, the minimum activity level which would provide a count of greater than the background count is 47.1 kBq ± 14.9 kBq. The margin for error is calculated from the error for the background count as this is much greater than that for the cps with the source in air. As the activity levels of alpha contamination which would be encountered in the field would be of a wide range of values, from Bq to GBq, this sensor shows the potential for stand-off detection in a number, but possibly not all, potential situations. However, other sensors in the UVTron range are available, and these experiments were carried out with the off-the-shelf set up without optimisation or alteration for the specific use.

As the UVTron is similarly unresponsive to ambient light when in a room illuminated by either or both commercial lighting and natural light, the UVTron has little issue from interference from other lighting sources when used indoors. Although the background count is negligible in such conditions, it will provide a limiting factor in the sensitivity of the UVTron for alpha detection applications when counts approach background levels due to a decrease in signal either from a less active source or increased distance between source and detector. This may be relevant in applications with a low source activity, but will be less so in decommissioning where activity levels may be significantly high.

In combination, the background count in different lighting conditions, the effect of distance, the activity level of the source, and the variation of count frequency over time will determine the minimum sensitivity of the detector in any given field condition.

It has also been shown that a flow of gas over an alpha source, which may be more easily deployed in the field, can increase the fluorescence detected. Xenon resulted in an increase of 52%, with P-10 (32%), neon (26%), and krypton (23%), also enhancing fluorescence. Nitrogen did not provide a significant increase. Further gas flow tests, including the verification of results by repetition of the experiments carried out to date are planned.

## Figures and Tables

**Table 1:** Table of gases.

Gas	Symbol	Purity	Approximate Flow Rate mL/min
Nitrogen	N_2_	N5.0	65
Xenon	Xe	N5.0	50
P10	10% CH4/90% Ar	±5%	60
Krypton	Kr	N5.0	55
Neon	Ne	CP grade	40

**Table 2:** Variation in average counts per second (cps) by gas in comparison to air.

	Air	Nitrogen	Xenon	P10 CH4 10%; Ar 90%	Neon	Krypton
Duration of experiment (s)	57,600	3888	4203	5073	3721	3550
Total counts	18,890	1322	2103	2201	1537	1436
Average cps	0.3280	0.3400	0.5004	0.4339	0.4131	0.4045
Counting Uncertainty	0.7%	2.8%	2.2%	2.1%	2.6%	2.6%
Average cps difference to air	-	0.012	0.1724	0.1057	0.0851	0.0765
Percentage increase from air	-	3.6%	52%	32%	26%	23%
